# Minicircle HBV cccDNA with a Gaussia luciferase reporter for investigating HBV cccDNA biology and developing cccDNA-targeting drugs

**DOI:** 10.1038/srep36483

**Published:** 2016-11-07

**Authors:** Feng Li, Liang Cheng, Christopher M. Murphy, Natalia J. Reszka-Blanco, Yaxu Wu, Liqun Chi, Jianming Hu, Lishan Su

**Affiliations:** 1 Lineberger Comprehensive Cancer Center, Department of Microbiology and Immunology, University of North Carolina at Chapel Hill, Chapel Hill, North Carolina 27599, USA; 2 Institute of Infectious Diseases, Guangzhou Eighth People’s Hospital, Guangzhou Medical University, Guangzhou, Guangdong Province 510060, China; 3 Department of Translational Medicine, Department of Surgery, Department of Medicine, the First Hospital, Jilin University, Changchun, Jilin Province 130061, China; 4 Department of Microbiology and Immunology, Penn State College of Medicine, Hershey, PA 17033-0850, USA.

## Abstract

Chronic Hepatitis B Virus (HBV) infection is generally not curable with current anti-viral drugs. Virus rebounds after stopping treatment from the stable HBV covalently-closed-circular DNA (cccDNA). The development of drugs that directly target cccDNA is hampered by the lack of robust HBV cccDNA models. We report here a novel HBV cccDNA technology that will meet the need. We engineered a minicircle HBV cccDNA with a Gaussia Luciferase reporter (mcHBV-GLuc cccDNA), which serves as a surrogate to measure cccDNA activity. The mcHBV-GLuc cccDNA was easily produced in bacteria, and it formed minichromosomes as HBV cccDNA episome DNA does when it was transfected into human hepatocytes. Compared to non-HBV minicircle plasmids, mcHBV-GLuc cccDNA showed persistent HBV-GLuc activity and HBx-dependent gene expression. Importantly, the mcHBV-GLuc cccDNA showed resistance to interferons (IFN) treatment, indicating its unique similarity to HBV cccDNA that is usually resistant to long-term IFN treatment in chronic HBV patients. Most importantly, GLuc illuminates cccDNA as a surrogate of cccDNA activity, providing a very sensitive and quick method to detect trace amount of cccDNA. The mcHBV-GLuc cccDNA model is independent of HBV infection, and will be valuable for investigating HBV cccDNA biology and for developing cccDNA-targeting drugs.

Although a preventive HBV vaccine is available, there is no cure for the350 million patients who are already chronically infected, often leading to liver cirrhosis and hepatocellular carcinoma (HCC)^1^,^2^. Clinically, nucleoside analogues (NUC) can effectively suppress HBV virus production by blocking the reverse transcription polymerase step, but they fail to eliminate the HBV cccDNA, which contributes to the rapid virus rebound after drug withdrawal^3^. Similarly, the emerging new therapeutics targeting HBV virus entry can only prevent new infection, but cannot eliminate the cccDNA pool per se in the infected cells^4^,^5^,^6^,^7^. NUC and IFNα are the only two types of FDA approved therapeutics^1^,^8^. IFNα treatment can sometimes achieve long-lasting suppression through a poorly understood mechanism^9^, but sustained virological response and HBsAg loss is achieved in only a small fraction (3–7%) of patients^10^ after 48-week (PEG)-IFNα treatment, which requires high costs and significant side effects. HBV DNA couldn’t be cleared even when seroconversion was achieved^11^. Altogether, the current treatments can only suppress the production of new HBV viremia, but cannot clear HBV cccDNA and cure chronic HBV.

The mechanism of HBV cccDNA generation and maintenance is still not elucidated. Reports based on studying the Duck Hepatitis Virus (DHBV) show that cccDNA binds nucleosome proteins and forms a minichromosome^12^, which can be stably maintained in nondividing hepatocytes during treatment as an episomal DNA^13^. The HBV viral rebound was observed even after decades of successful HBV suppression associated with robust anti-HBV immune response after acute infection^14^,^15^,^16^,^17^ or after IFNα treatment^11^, indicating that episome cccDNA can persist stably in an occult status. As minichromosomes, cccDNA has been identified to be associated with histone proteins and its function is regulated by epigenetic “writers”, “readers” and “erasers”^18^,^19^,^20^,^21^,^22^,^23^. However, the structure and the regulation of HBV cccDNA minichromosome is still unclear. One reason is the very low copy number of HBV cccDNA in the available infection models such as primary human hepatocytes, HepaRG^24^ and HepG2-NTCP cells^25^,^26^, and in HepG2cell lines stably producing HBV^27^,^28^,^29^. The other reason, even more important, is the lack of sensitive and reliable methods to measure cccDNA and especially its specific activity^27^,^28^,^29^,^30^. To accurately measure HBV cccDNA in infected cells, PCR-based assay is extremely challenging because of the vast excess of non-cccDNA replication intermediate^30^. The Southern Blot assay to detect cccDNA is less sensitive^27^,^28^,^29^, and technically challenging for high-through-put application. A better understanding of the structure and function of cccDNA will benefit the development of novel therapeutics to target cccDNA and cure HBV infection. Therefore, a robust HBV cccDNA-based system is urgently needed to investigate the HBV cccDNA biology and to speed up anti-HBV cccDNA drug development^31^,^32^.

In this study, we report the development of a novel HBV cccDNA technology that will overcome the limitations. We engineered the HBV-GLuc virus to its cccDNA form, a minicircle HBV-GLuc (mcHBV-GLuc) cccDNA easily produced in bacteria, which can function as HBV cccDNA when transfected into human hepatocyte cells. Importantly, the sensitive GLuc reporter illuminates the cccDNA, serving as a quick measurement of cccDNA activity. Collectively, the new HBV cccDNA technology will provide a useful platform both for high throughput screening of new drugs to inhibit and/or eliminate HBV cccDNA, and for studying HBV and its host interaction.

## Results

### Generation of minicircle HBV-GLuc cccDNA (mcHBV-GLuc cccDNA)

**Figure 1:**
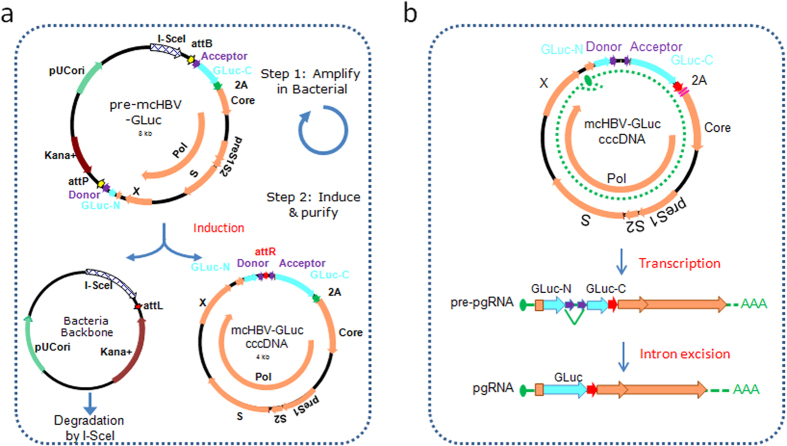
Generation of a novel functional mcHBV-GLuc cccDNA model. (**a**) Strategy to generate mcHBV-GLuc cccDNA in *E. coli*. The pre-mcHBV-GLuc in the plasmid backbone can be amplified in *E. coli*. After recombinase induction, pre-mcHBV-GLuc is subjected to attP and attB recombination and produces two DNA circles. One with the bacteria backbone (left)is degraded by the I-SceI, while the other (right) mcHBV-GLuc cccDNA is maintained. pUCori: plasmid replication origin; Kana + : kanamycin resistance gene coding sequence; I-SceI: tandem I-SceI cutting sites; attB and attP: recombinase recognizing sites; attL and attR: sequences generated by attB and attP recombination; Donnor and Acceptor: Splicing donor and acceptor sequences; GLuc-N and GLuc-C: N and C terminal of GLuc coding sequences; 2A, Core, Pol, PreS1/S2/S, X are as in Fig. 1a. (**b**) GLuc expression from mcHBV-GLuc cccDNA. GLuc gene (blue) is split into GLuc-N and GLuc-C by an intron (purple, with splicing donor and acceptor sequence). Pre-pregenomic RNA (Pre-pgRNA) transcript is generated from mcHBV-GLuc cccDNA. The intron is then spliced out and GLuc-N and GLuc-C are ligated to form the functional Gluc mRNA.

HBV has a very compacted genome. The whole HBV genome sequence is used for coding viral proteins and all the transcription regulatory elements are embedded in coding genes. It has been extremely challenging to insert any exogenous DNA sequence to its highly compact and efficiently utilized genome without inactivating HBV infectivity. We engineered a minicirlce HBV-GLuc cccDNA vector by inserting a GLuc reporter gene in the Core sequence (Fig. 1a). We chose the Core 5′ region for GLuc insertion for the following reasons: (1) the Core 5′ region has only one open reading frame (ORF) and is reported to contain no known regulatory sequence; and (2) the Core expression is from pgRNA which is transcribed from cccDNA using the HBV core promoter. To make the GLuc expression from pgRNA, GLuc was split in the middle and inserted with 1.0 fold HBV genome into a minicircle vector to get a pre-mcHBV-GLuc cccDNA (Fig. 1a)^33^. As shown in Fig. 1a, the GLuc cannot be expressed from the pre-mcHBV-GLuc cccDNA because of the DNA sequences separating GLuc-N and GLuc-C. The pre-mcHBV-GLuc cccDNA can replicate and be amplified in bacteria using its replication origin^33^.

The mcHBV-GLuc cccDNA is produced using a ØC31-mediated DNA recombination system in which the bacterial plasmid DNA backbone was eliminated and degraded (Fig. 1a). Large quantity of mcHBV-GLuc cccDNA can be produced with high purity^33^. However, an attR sequence will be a residue in mcHBV-GLuc cccDNA after the recombination, which disrupted the GLuc reading frame, and prevented from producing full length GLuc and Core. To overcome the limitation, a pair of intron splicing and donor sites was introduced to flank the attR site (Fig. 1b). And a synthesized sequence with synonymous codons was used to restore the missing 5′ terminus of Core. The 2A protein self-cleavage sequence was inserted between GLuc and Core to express both GLuc and Core as used in our influenza GLuc reporter virus^34^. As illustrated in the lower part of Fig. 1b, when the mcHBV-GLuc cccDNA is transfected into human hepatocyte cells, the pre-pgRNA is transcribed, but still couldn’t produce GLuc and Core protein because of the open reading frame is disrupted by the inserted intron. A functional pgRNA is obtained to produce GLuc and Core proteins only after the intron is spliced out of pre-pgRNA. Therefore, mcHBV-GLuc cccDNA is easily produced in bacteria using minicircle technology, and the intron sequence guarantees GLuc and Core are both expressed from the mcHBV-GLuc cccDNA. The GLuc signal comes from pgRNA, providing an easy and quick surrogate of cccDNA activity.

### The intron sequence is efficiently spliced to produce full length GLuc and Core proteins

**Figure 2:**
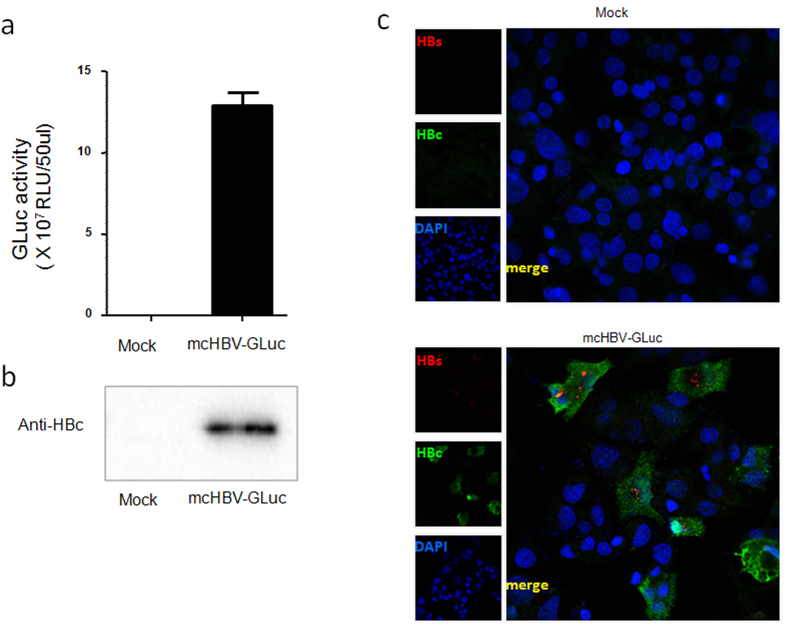
The intron is efficiently spliced to express GLuc and Core from mcHBV-GLuc cccDNA in HepG2 cells. (**a**) GLuc expression in the supernatant of HepG2 cells transfected with mcHBV-GLuc cccDNA at 3 day post transfection. **(b)** Core expression was detected in HepG2 cells transfected with mcHBV-GLuc cccDNA using western blot (cropped, run in the same gel and in the same experimental conditions). (**c**) Co-expression of HBc and HBs in transfected cells. HepG2 cells were transfected with mcHBV-GLuc cccDNA for 5 days, fixed and stained with anti-HBc (green) and anti-HBs antibody (red). RLU, relative light units. Data are means ± s.e.m. from triplicate wells and are representative of three independent experiments. Mock: HepG2 cells transfected with pCDNA control. mcHBV-GLuc: HepG2 transfected with mcHBV-GLuc cccDNA.

The intron splicing as designed was tested in HepG2 cells. HepG2 cells were transfected with mcHBV-GLuc cccDNA. At 3 day post transfection, high levels of secreted GLuc (2.6e9RLU/ml) were detected in the medium (Fig. 2a) indicating that the intron was accurately spliced. In addition, mature core protein was detected by western blot in transfected cells (Fig. 2b), indicating that the 2A could efficiently separate lead to expression of both GLuc and Core as in our previous influenza study^34^ (and data not shown). We then tested the splicing efficiency of the intron by using independently expressed HBs as an internal control. Transfected cells were co-stained with anti-HBc and anti-HBs expression (Fig. 2c). All HBs positive cells (Red) were also HBc positive (Green), suggesting that the intron was efficiently spliced in most if not all cells.

### The mcHBV-GLuc cccDNA produces DNA containing viral particles in human hepatocyte cells

**Figure 3:**
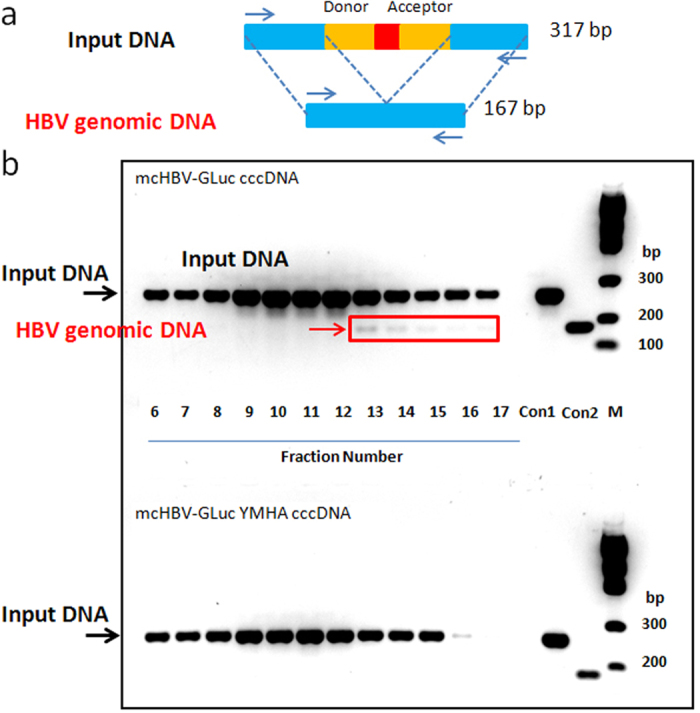
mcHBV-GLuc cccDNA produces virion-associated HBV genome from transfected human cells. Huh7.5 cells were transfected with mcHBV-GLuc cccDNA and mcHBV-GLuc YMHA cccDNA (a mutant with defective reverse transcriptase activity). The viruses in the culture supernatant were concentrated with 6% PEG8000, and fractioned with idixanol gradient centrifugation. The fractions from 6 to 17 were analyszed with PCR primers covering the Intron. (**a**) A 317bp product was detected using the input mcHBV cccDNA as template, and a smaller 167bp product was detected from virion-associated HBV genome. (**b**) HBV viron-associated HBV genome was detected in fractions 13 to 17 in the transfection with mcHBV-GLuc cccDNA (arrow and box), but was undetectable in the transfection with the mcHBV-GLuc YMHA cccDNA mutant DNA. Con 1: PCR product with intron sequence using mcHBV cccDNA plasmid as template. Con 2: PCR product using HBV-GLuc plasmid without intron sequence as template. M: DNA ladder, 300, 200 and 100 bp are shown.

Next, we determined whether the mcHBV-GLuc cccDNA could produce DNA containing viral particles. Huh7 cells were transfected with mcHBV-GLuc cccDNA and mcHBV-GLuc YMHA cccDNA with inactivated reverse transcriptase activity^35^. Secreted HBV viral particles were purified by PEG8000 concentration and iodixanol grandient centrifugation^36^. Specific PCR primers spanning the intron (Fig. 3a) were used to detect whether spliced form of viral genome DNA was generated. As expected, HBV genomic DNA without intron was detected from transfected mcHBV-GLuc cccDNA in fraction 13, but not from mcHBV-GLuc YMHA (RT polymerase mutant) cccDNA, indicating that the mcHBV-GLuc cccDNA could produce HBV-GLuc viruses (Fig. 3b). However, the GLuc insertion made the HBV genome too large and comprised the virus yields (Li and Su unpublished data).

### The mcHBV-GLuc cccDNA shows similar chromatinization and activity of HBV cccDNA

**Figure 4:**
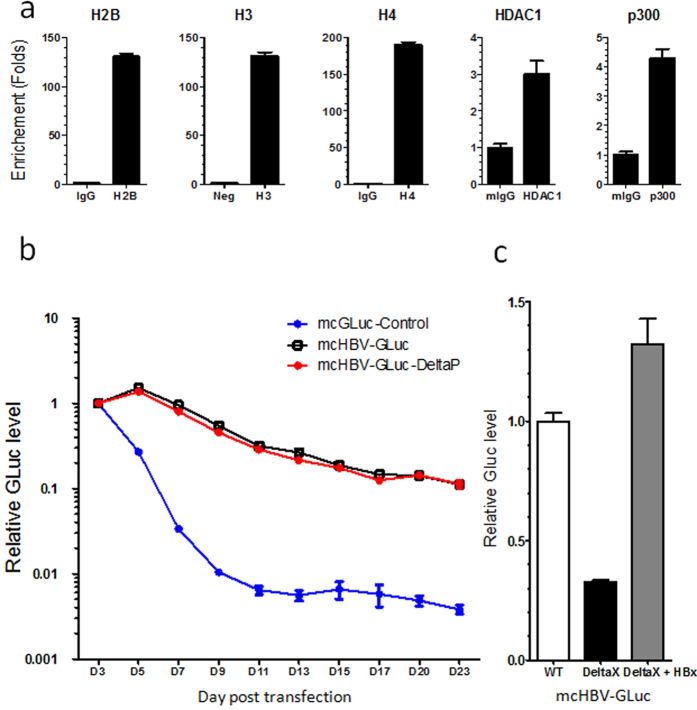
mcHBV cccDNA forms minichromosome and stably expresses GLuc in anHBx-dependent manner. (**a**) ChIP analysis of the mcHBV-GLuc cccDNA minichromosome in HepG2 cells at day6 after transfection. Antibodies against H2B, H3, H4, HDAC1and p300 were used for ChIP. Quantitative PCR with specific primers were used to measure the HBV genome copy number. Relative enrichment (fold) was calculated relative to their respectively isotype IgG controls. (**b**) Persistent GLuc activity in HepG2 cells transfected with mcHBV-GLuc cccDNA. HepG2 cells were transfected with mcHBV-GLuc cccDNA, mcHBV-GLuc-DeltaP cccDNA or non-HBV minicircle control DNA (mcGLuc-Control, Supplemental Fig. 1). Relative GLuc levels were calculated by normalizing GLuc titers to the level on day 3 of each group. (**c**) HBx enhances GLuc expression from mcHBV-GLuc DeltaX cccDNA. WT: wild type mcHBV-GLuc cccDNA, DeltaX:mcHBV-GLuc cccDNA without HBx expression, HBx: HBx-expressing plasmid. Data are representative of three independent experiments.

Due to the very low aboudance of cccDNA in the HBV infection, the knowledge about HBV cccDNA is limited. The HBV cccDNA is reported to exist as an episome in the form of a minichromosome^12^ with stable persistence^24^. The HBV-encoded HBx is critical for HBV cccDNA gene expression^19^,^37^. To determine whether the mcHBV-GLuc cccDNA can form minichromosomes in mammalian cells, we applied chromatin immune precipitation (ChIP) to detect the association of chromatin components to HBV cccDNA. In HepG2 cells at day 6 post transfection with mcHBV-GLuc cccDNA, HBV cccDNA was co-IPed with antibodies against histone proteins, such as H2B, H3, and H4 (more than 100 times over isotype control) and with antibodies against HDAC1 and p300^38^ (Fig. 4a), indicating that transfected mcHBV-GLuc cccDNA formed minichromosomes. We next tested persistent activity of mcHBV-GLuc cccDNA, the GLuc activity in supernatant of HepG2 cells transfected with mcHBV-GLuc cccDNA was monitored for over 3 weeks. As a control, the activity of the non-HBV control minicircle DNA mcGLuc (Supplementary Fig. 1a) decreased rapidly by 100 fold in 7–9 days (Fig. 4b). The activity of mcHBV-GLuc cccDNA, however, showed only a minor decrease over 23 days. To determine whether the persistent GLuc activity came from the input mcHBV-GLuc cccDNA or from secondary, newly formed HBV-GLuc cccDNA, mcHBV-GLuc-DeltaP cccDNA, a Pol deletion mutant, was used as a mcHBV-GLuc cccDNA replication deficient control (Supplementary Fig. 1b,c). No difference between the mcHBV-GLuc cccDNA and mcHBV-GLuc-DeltaP-cccDNA was detected in its stability (Fig. 4b), suggesting that the long GLuc persistence is not due to newly-formed cccDNA but from transfected mcHBV-GLuc cccDNA. As shown above, the GLuc insertion greatly comprised the generation of cccDNA from input mcHBV-GLuc cccDNA. Finally, we tested whether the pgRNA was controlled in an HBx-depenpent manner, a very important feature of cccDNA as previously reported^19^,^37^,^39^. HBx deletion mutant mcHBV-GLuc-DeltaX cccDNA was made by introducinga stop codon at the 8^th^ amino acid (Gln (CAA) to stop (TAA) as reported^37^,^39^. The mcHBV-GLuc-DeltaX cccDNA was transfected into a Tet controlled HepG2-HBx cell line. The GLuc activity was compared in the presence (HBx+) and absence (HBx−) of Dox (Fig. 4c). HBx provided in trans greatly increased the GLuc expression by 4–5 folds, indicating that mcHBV-GLuc cccDNA is functionally similar to HBV cccDNA generated during HBV infection in its regulation of pgRNA transcription. Therefore, the mcHBV-GLuc cccDNA can recapitulate the characteristics of HBV cccDNA, and the Gluc expression can serve as a surrogate for quickly measuring cccDNA activity.

### mcHBV-GLuc cccDNA is resistant to interferons in comparison to other minicircle non-HBV DNA, or the pre-mcHBV-GLuc DNA with bacterial plasmid sequences

**Figure 5:**
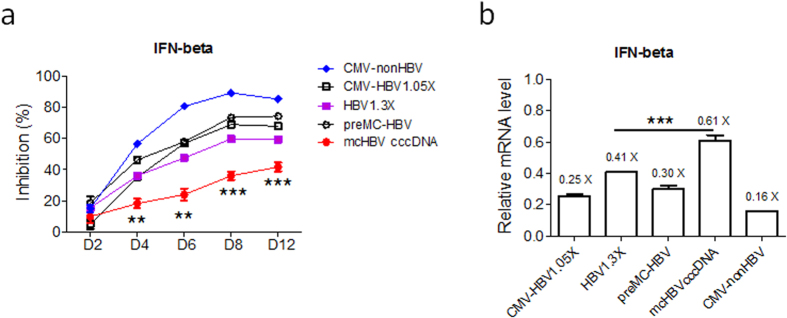
mcHBV cccDNA is resistant to IFN treatment. (**a**) HepG2 cells transfected with different plasmids were treated with IFN-beta (500 U/ml) for 4 days at 2 days post transfection. The inhibition of Luciferase gene expression relative to mock treatment was calculated at day 2, 4, 6, 8 and 12 post IFN treatment (D2, D4, D6, D8, and D12). The relative mRNA levels at 12 days post treatment were measured by RT-PCR (**b**). HBV1.3X:pHBV-1.3X-GLuc, preMC-HBV: pre-mcHBV-GLuc, and mcHBV cccDNA: mcHBV-GLuc cccDNA. CMV-HBV1.05X: pCMV-HBV1.05X-GLuc, CMV-nonHBV: non HBV plasmid pCLucIPZ. **p < 0.01, ***p < 0.001.

HBV cccDNA is speculated to be highly resistant to IFN treatment in CHB patients. To test if the mcHBV-Gluc cccDNA is similarly resistant to interferons, HepG2 cells transfected with mcHBV-Gluc cccDNA or its pre-minicircle form, as well as commonly used HBV genome DNA constructs in bacterial plasmids, were treated with IFN-beta (500 U/ml), and the inhibitory effect was calculated (Fig. 5a). IFN-beta effectively inhibited the activity of non-HBV genes (CMV-nonHBV), HBV full genome in bacterial plasmids (CMV-HBV1.05X and HBV1.3X, Supplementary Fig. 2) and the pre-minicircle mcHBV cccDNA plasmids (preMC-HBV), but the mcHBV cccDNA showed significant resistance (Fig. 5a). The IFN-beta inhibitory effect was also confirmed by measuring the relative mRNA level (Fig. 5b). While the IFN treatment didn’t result in the lost of DNA (Supplementary Fig. 3). The results above suggest that the mcHBV cccDNA is functionally distinct from both those plasmids with bacterial DNA backbones and minicircle non-HBV DNA in relative resistance to IFN induced inhibition.

### The mcHBV-GLuc cccDNA provides a high throughput screening for cccDNA-targeting drugs

**Figure 6:**
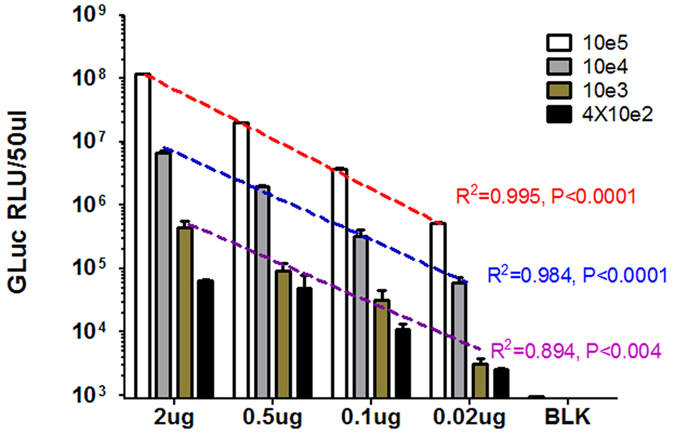
Titration of cell number and mcHBV-GLuc cccDNA plasmid dose in HepG2 cells. 1 million HepG2 cells were nucleofected with 2 ug, 0.5 ug, 0.1 ug, 0.02 ug of mcHBV-GLuc cccDNA (H-022 program). Cell concentration was then diluted to 10e5, 10e4, 10e3 and 4 × 10e2 cells/well in 96 well plates. And the GLuc activity was measured at day 3 post transfection. Data was mean ± s.e.m of duplications. BLK: blank control with no transfection.

Our major goal is to develop a highly efficient platform to screen drugs directly targeting cccDNA. The system was then optimized to test what’s the best condition for drug study. HepG2 cells were first transfected with different amount of mcHBV-GLuc cccDNA (2 ug, 0.5 ug, 0.1 ug and 0.02 ug in 10e6 cells) using Amaxa nucleofector and were diluted to 10e5, 10e4, 10e3, 4 × 10e2 cells in 96-well plates. The GLuc in the supernatant was detected at day 3 post transfection (Fig. 6). Good linear correlation was observed with 10e5, 10e4, 10e3 cells/well. Remarkably, even 1000 cells/well in the 0.02 ug group showed a significant higher GLuc signal above blank (BLK) control, indicating the high sensitivity of the GLuc as a surrogate reporter of the HBV cccDNA activity.

## Discussion

We report here a novel and sensitive strategy to mimic the function of HBV cccDNA. The mcHBV-GLuc cccDNA showed the characteristics of bono fide HBV cccDNA^12^,^13^. First, we demonstrate that mcHBV-GLuc cccDNA formed minichromosome in transfected cells, a key feature of cccDNA, by binding to known histone proteins and histone associated proteins (Fig. 4a)^18^,^19^,^20^,^21^,^22^,^23^,^38^,^40^. Second, the mcHBV-GLuc cccDNA remained active for more than three weeks in HepG2 cells (Fig. 4b), in contrast to non-HBV minicircle DNA or control plasmid HBV genomic DNA. It has been reported that HBV cccDNA persists stably in chronic infected patients through unknown mechanism(s) even when virus replication is effectively suppressed for decades^11^,^14^,^15^,^16^,^17^. HBV cccDNA can also be maintained in HBV-infected HepaRG cells for several weeks^24^. Third, the HBV regulatory protein HBx is known to activate cccDNA transcription through an unclear mechanism^19^,^32^,^37^,^39^. We observed that Gluc or pgRNA transcription from the mcHBV-GLuc cccDNA was efficiently up-regulated *in trans* by HBx (Fig. 4c). Finally, but most importantly, we demonstrate that the mcHBV-GLuc cccDNA resisted IFN-beta treatment (Fig. 5), consistent with reported resistance of HBV cccDNA to IFNs in HBV infection models^20^,^41^. Therefore, the mcHBV-GLuc cccDNA is functionally similar to HBV cccDNA.

In addition to its similarity to HBV cccDNA, mcHBV-GLuc cccDNA has the following advantages. First, the mcHBV-GLuc cccDNA can be easily generated in bacteria and transfected into any cell types. Second, the GLuc is under control of HBV core promoter and HBV core ATG in the pgRNA, providing a quick and sensitive measurement of HBV cccDNA activity, and making it possible to distinguish functional HBV cccDNA from other forms of HBV DNA, such as integrated HBV, rcDNA, and dslDNA. HBV pgRNA is not only the template for reverse transcription to generate genomic DNA, but also is translated to produce both core and viral polymerase proteins. However, measurement of pgRNA is limited to pgRNA detection either with Northern Blot or other RNA based techniques, which are difficult for high throughput screening. Finally, the mcHBV-GLuc cccDNA makes it possible to genetically study HBV cccDNA to define the determinants for it stability and its host interactions, which will lead to discovering new targets and exploring new strategies to inhibit cccDNA, and ultimately to cure HBV infection.

Lastly, the mcHBV cccDNA is different from authentic cccDNA because it is produced in bacteria and transfected into HepG2 cells. Interestingly, the mcHBV cccDNA showed persistent activity for at least 3 weeks post transfection (Fig. 4b). We hypothesize that the mcHBV cccDNA will be well modified by host factors to form cccDNA-like minichromosome (Fig. 4a). Some of the key findings will be confirmed with HBV cccDNA in HBV-infected cells in future experiments.

The HBV-GLuc cccDNA technology will be invaluable in studying HBV cccDNA biology and in screening for cccDNA targeting drugs. Besides GLuc, other reporter genes can be employed for different purposes. It should be noted that this strategy is not restricted to HBV. Other DNA viruses with a circular genome, including polyomaviruses and papillomaviruses, could also be investigated using this strategy.

Note: While our manuscript was under review, a new independent paper describing a similar but different cccDNA system was published (Sci Rep. 2016 May 13;6:25552, ref. 42). In their system, the HBx coding region (also the Core promoter region) was manipulated for producing minicircle HBV cccDNA but with no reporter genes. Our system differs from their system in the following two aspects: 1) We used the HBc coding region to insert foreign DNA sequence and employed an intron to regulate gene expression; and 2) we included a GLuc reporter gene for measuring cccDNA activity from the pg RNA promoter, which can be potentially used for high throughput screening.

## Materials and Methods

### Plasmids

HBV genome was cloned from a HBV patient serum (Genotype C2) using a modified method, the Gluc-2A^34^ was amplified using overlapping PCR, and inserted into the Core region in the HBV genome. To construct the pre-mcHBV-GLuc, a long segment including the splicing acceptor sequence (from pSI, Promega) (cacctattggtcttactgacatccactttgcctttctctccacag), the whole HBV-Gluc genome and the donor sequence (from pSI, Promega) (gtaagtatcaaggttacaagacaggtttaaggagaccaatagaaa) was amplified using several rounds of overlapping PCR and was used to replace EF1a-MCS-SV40polyA fragment in the pMC.EF1-MCS-SV40polyA(MN502A-1, System Biosciences). Based on the pre-mcHBV-GLuc cccDNA, point mutations were introduced to get pre-mcHBV-GLuc-DeltaP, pre-mcHBV-GLuc-DeltaXand pre-mcHBV-GLuc-YMHA. Control plasmid mcGLucwas generated by insert EGFP-GLuc fragment into pMC.EF1-MCS-SV40polyA. pCLucIPZ was obtained from Addgene (Cat: 53222). pGFP-HBx from Addgene (Cat: 24931). Lenti-G9a plasmid is obtained from UNC lentivirus vector core facility. pCMV-HBV1.05X-GLuc and pHBV1.3X-GLuc were generated by inserting the GLuc reporter gene into the HBV Core ORF, and GLuc expression is under control of CMV and HBV Core promoter, respectively. Phusion High-Fidelity DNA Polymerase (Thermo Scientific) was used for all the PCR reactions. Q5^®^ Site-Directed Mutagenesis Kit (NEB) was used for the point mutations. And all the restrictive enzymes were from Thermo Scientific. And the T4 DNA ligase was from NEB.

### Cell culture and transfection

293T, HepG2, HepG2-NTCP-C4 and HepG2-CLuc cells were maintained in DMEM containing 10% FBS, 1X PS, 1X HEPES, 1X L-Glu, 1X NEAA, 1X Pyruvate sodium (Invitrogen). HepG2-CLuc stable cells were generated by infecting HepG2 cells with CLucIPZ lentivirus generated from 293T cells. Two protocols were used for transfecting HepG2 and HepG2-Cluc cells. (1) Electroporation was used to transfect using Amaxa cell line nucleofectoraccording to manufacturer’s instructions (Lonza,AmaxaNucleotransfection Kit V,and program H-022). (2) Lipofectamine3000 (Invitrogen) transfection was used with a modified reverse transfection protocol. Briefly, 10 ug total DNA was mixed with lipofectamine3000. Thencells were treated with trypsin (0.25%, Invitrogen) and single cells were made. Finally, 6–10 million cells were suspended with DNA liposome and plated for one 10-cm plate. Recombinant human IFN-beta 1a (R&D, Catalog #11410-2) was used to treat HepG2 cells transfected with different plasmids.

### Chromatin Immunoprecipitation Assays

The cccDNA ChIP assays were performed as described in the kit protocol (Magna ChIP TM A/G, Millipore), with minor modifications. Nuclei isolation from mouse liver followed the protocol^43^ with Dounce homogenizer and fixed with. The nuclear sonification was carried out using the sonicator Covaris E220 at 4 °C with two rounds of 5 mins sonification +5 mins interval, and one round of 3 mins sonification. Cross linked chromatin was subjected to immunoprecipitation for 15–20 h at 4 °C in a rotor. Antibodies specific to H2B (Millipore, Catalog # 17-10054), H3 (Millipore, Catalog # 17-10046), H4 (Millipore, Catalog # 17-10047), p300 (Santa Cruz, Catalog # sc-585), pCAF (Millipore, Catalog # 17-10532), HDAC (Millipore, Catalog # 17-608), and the relevant negative control are used. Immunoprecipitated chromatins were processed and analyzed by quantitative PCR using HBV core primers.

### Luciferase measurement

10–50 ulof culture medium were used to measure GLuc (*Renilla* Luciferase Assay System, Promega) and CLuc (BioLux^®^ Cypridina Luciferase Assay Kit, NEB). The relative GLuc activity was normalized to CLuc.

### HBV DNA and RNA detection

Serum and cell culture HBV viral genomic DNA was prepared with QIAampMinElute Virus Spin Kit (Qiagen). Total cellular genomic DNA was prepared using DNeasy Blood & Tissue Kit (Qiagen). Total RNA was prepared from HepG2 or HepG2-CLuc cells using RNeasy Plus Mini Kit (Qiagen). Then, the cDNA was generated using reverse transcriptase III (Invitrogen). HBV mRNA was quantified using ABsolute QPCR Mix, SYBR Green, ROX(Thermo Scientific). Primers HBc-F (GAGTGTGGATTCGCACTCC) and HBc-R (GAGGCGAGGGAGTTCTTCT) were used to measure HBV genomic DNA and pgRNA. HBx-F (TCACCAGCACCATGCAAC) and HBx-R (AAGCCACCCAAGGCACAG) were used to measure the HBV total mRNA. Q-beta-actin-F (CCATCATGAAGTGTGACGTGG) and Q-beta-actin-R (GTCCGCCTAGAAGCATTTGCG) were used to normalize the cellular total mRNA. CD4-F (CACGGGGAGTCAGCACCTTAT) and CD4-R (GGGATATGGCATCACAGCCT) were used to normalize the cellular genomic DNA.

### Immunofluorescence staining

Mock HepG2 and HepG2 cells transfected with mcHBV-GLuc cccDNA were seeded on the glass cover in 24-well plates. At day 3 post transfection, cells on the slides were fixed with 4% paraformaldehyde, permeabilized and blocked with 0.1% Triton X-100, 0.1% BAS and 5% goat serum in PBS. Cells were then incubated with Anti-HBcAg antibody (Zeta Corp) and Anti-HBsAg antibody (Abcam) for 1 h at room temperature, washed, and incubated with secondary antibodyfor 1 h: Alexa Fluor 488 Donkey Anti-Rabbit IgG (Life Technologies), and Alexa Fluor 594 Donkey Anti-Mouse IgG (Life Technologies). Cells were mounted with anti-fade mounting media with DAPI (Abcam Cambridge, MA; 104139). Images were collected with Zeiss LSM 700 Confocal Laser Scanning Microscope.

### Western blot

Adherent cells were washed with ice-cold PBS twice and scraped off. Cell pellets were lysed in radioimmunoprecipitation assay (RIPA) buffer (50 mMTris (pH 8.0), 0.1% SDS, 150 mMNaCl, 1% Nonidet P-40, 0.5% sodium deoxycholate and protease inhibitor cocktail), sonicated for 30 seconds, and then subjected to SDS-PAGE. Immunoblotting was performed using anti-HBc (Zeta Corp), anti-HBxAg (Abcam), anti-beta-Actin (Life technology). Relative band intensity was calculated using ImageJ software (NIH).

### Statistical Analysis

Statistical analysis was performed using Students’ t-test and the P-value were calculated. The correlation was analysis using GraphPad software, and the R^2^ and P-values were calculated.

## Additional Information

**How to cite this article**: Li, F. *et al.* Minicircle HBV cccDNA with a Gaussia luciferase reporter for investigating HBV cccDNA biology and developing cccDNA-targeting drugs. *Sci. Rep.* **6**, 36483; doi: 10.1038/srep36483 (2016).

**Publisher’s note**: Springer Nature remains neutral with regard to jurisdictional claims in published maps and institutional affiliations.

## Supplementary Material

Supplementary Information
