# Supplementary material for: Minicircle HBV cccDNA with a Gaussia luciferase reporter for investigating HBV cccDNA biology and developing cccDNA-targeting drugs
Source: Sci Rep. 2016 Nov 7;6:36483. doi: 10.1038/srep36483 (PMC5098228; doi:10.1038/srep36483)
Supplement: Supplementary Information [file srep36483-s1.pdf]

Title: Minicircle HBV cccDNA with a Gaussia luciferase reporter for investigating HBV cccDNA biology and developing cccDNA-targeting drugs

Authors: Feng Li[1,2],Liang Cheng[1], Christopher M Murphy[1], Natalia J Reszka-Blanco[1], Yaxu Wu[1], Liqun Chi[1],Jianming Hu[4],and Lishan Su[1,3*]

[1]Lineberger Comprehensive Cancer Center, Department of Microbiology and Immunology, University of North Carolina at Chapel Hill, Chapel Hill, North Carolina 27599, USA

[2]Institute of Infectious Diseases, Guangzhou Eighth People's Hospital, Guangzhou Medical University, Guangzhou, Guangdong Province 510060, China

[3]Department of translational medicine, Department of surgery, Department of medicine, the first hospital, Jilin University, Changchun, Jilin Province 130061, China

[4]Department of Microbiology and Immunology, Penn State College of Medicine, Hershey, PA 17033-0850

***Correspondence:** Correspondence should be addressed to F.L. &L.S. (lsu@med.unc.edu), telephone: 919-966-6654; fax: 919-966-8212

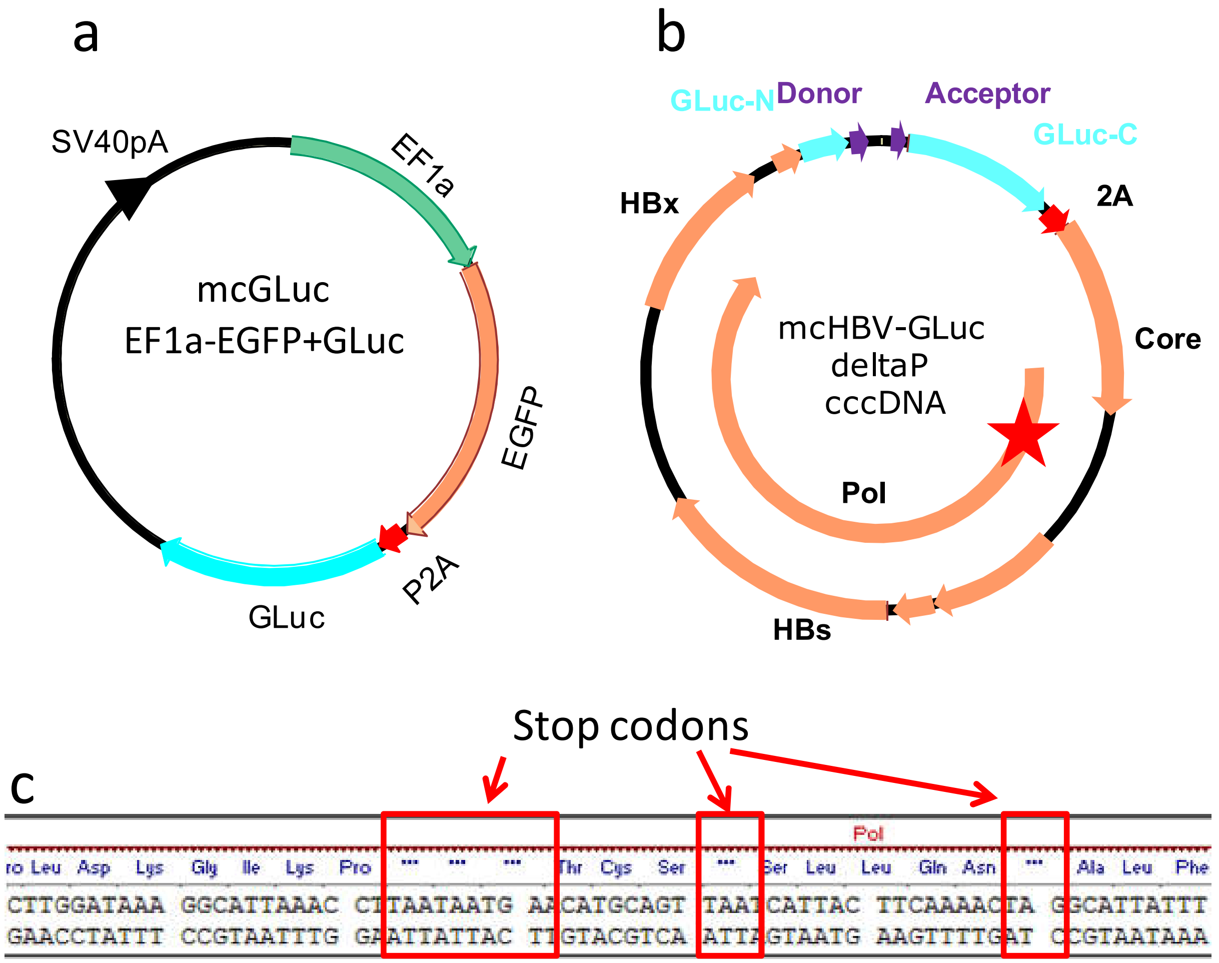

Supplementary Figure 1. Maps of minicircle DNAs used in main text. (a) mcGLuc control minicircle DNA. The EF1a promoter, EGFP-2A-GLuc open reading frame and SV40 poly A transcription termination sequence (SV40pA) are shown. (b) mcHBV DeltaP replication deficient minicircle DNA. Labels are as in Fig. 1a. Red star ★ indicates the position of Pol pre-termination. (c) The 5 stop codons introduced in tandem at the 5' coding region. Stop codons "…" are indicated in the red boxes.

Supp Figure 2 Li, et al

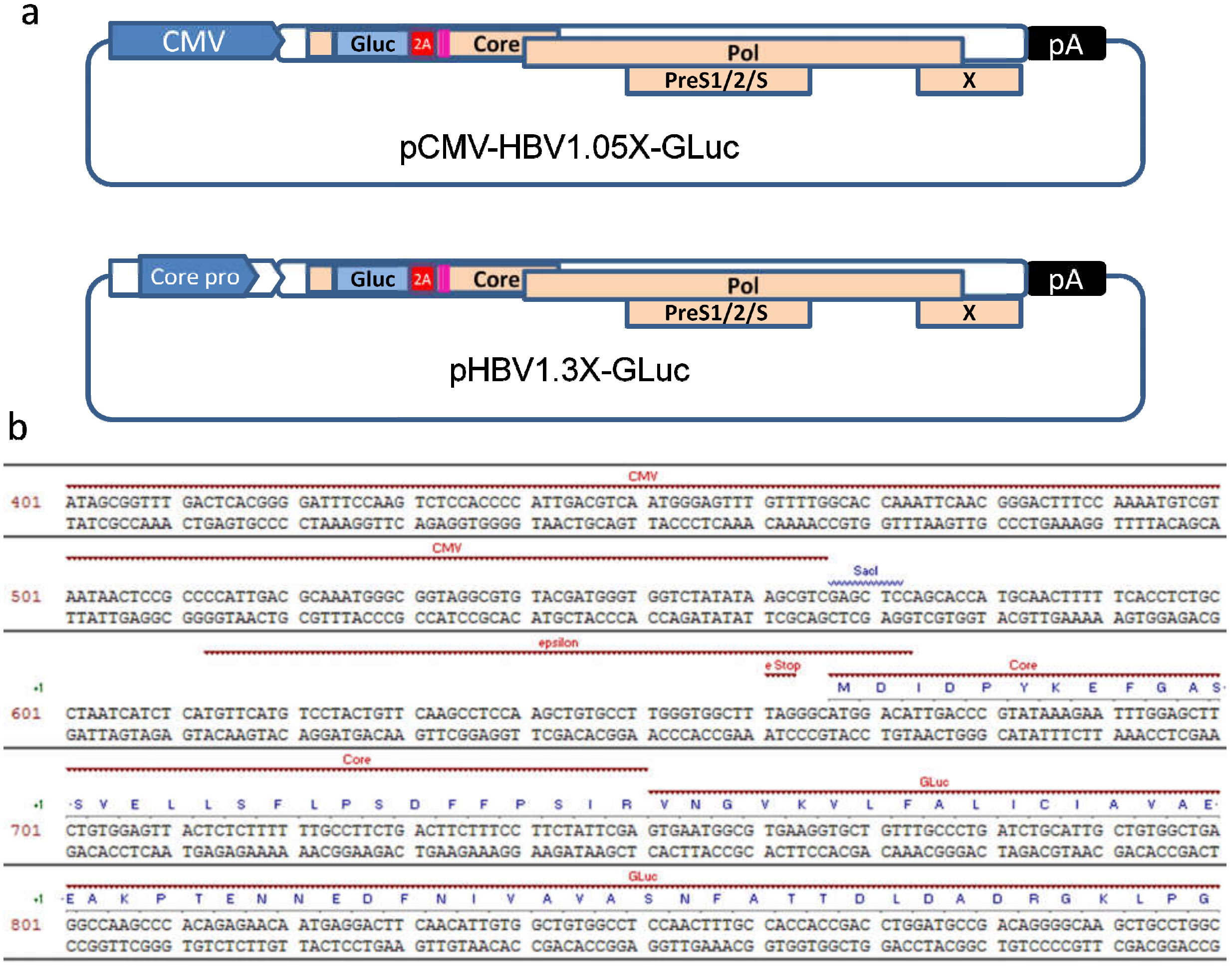

Supplementary Figure 2. Structure of HBV-Gluc plasmids. (a) GLuc was inserted into the Core coding region in the N terminal in pCMV-HBV1.05X-GLuc and pHBV1.3X-GLuc plasmids. Meanwhile, to guarantee the full length of Core protein, a synthesized sequence (purple in figure) which codes the very N terminal was added post 2A sequence. Full size Core protein is expressed. (b) The location for GLuc insertion in pCMV-HBV1.05X-GLuc is shown. CMV promoter (partial,CMV) is shown. The GLuc (partial) without ATG is inserted into the Core region (84bp from A (+1)of ATG). eStop is the HBe 1896 stop mutation, which prevents the production of HBeAg protein. SacI: cloning site.

Supp Figure 3 Li, et al

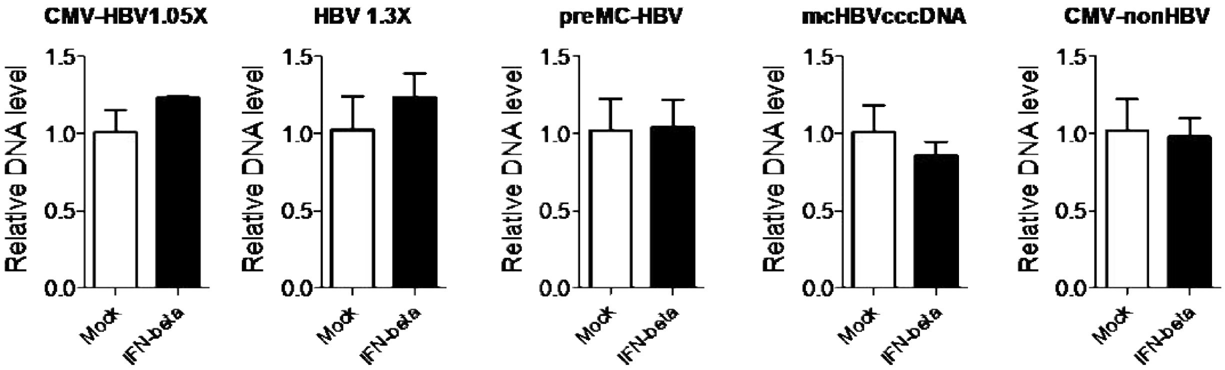

Supplementary Figure 3. The relative DNA levels at 12 days post IFN-beta treatment. HepG2 cells transfected with different plasmids were treated with IFN-beta (500U/ml) at 2 days post transfection. The relative DNA levels were normalized to Mock treated cells. HBV1.3X:pHBV-1.3X-GLuc, preMC-HBV: pre-mcHBV-GLuc, and mcHBV cccDNA: mcHBV-GLuc cccDNA. CMV-HBV1.05X: pCMV-HBV1.05X-GLuc, CMV-nonHBV: non HBV plasmid pCLucIPZ.
